# Supplementary figures and images for: The neuropeptide neuromedin U receptor NMUR-1 buffers insulin receptor signaling in bacteria-dependent C. elegans survival
Source: PLoS Genet. 2026 Jun 11;22(6):e1012190. doi: 10.1371/journal.pgen.1012190 (PMC13289896; doi:10.1371/journal.pgen.1012190)

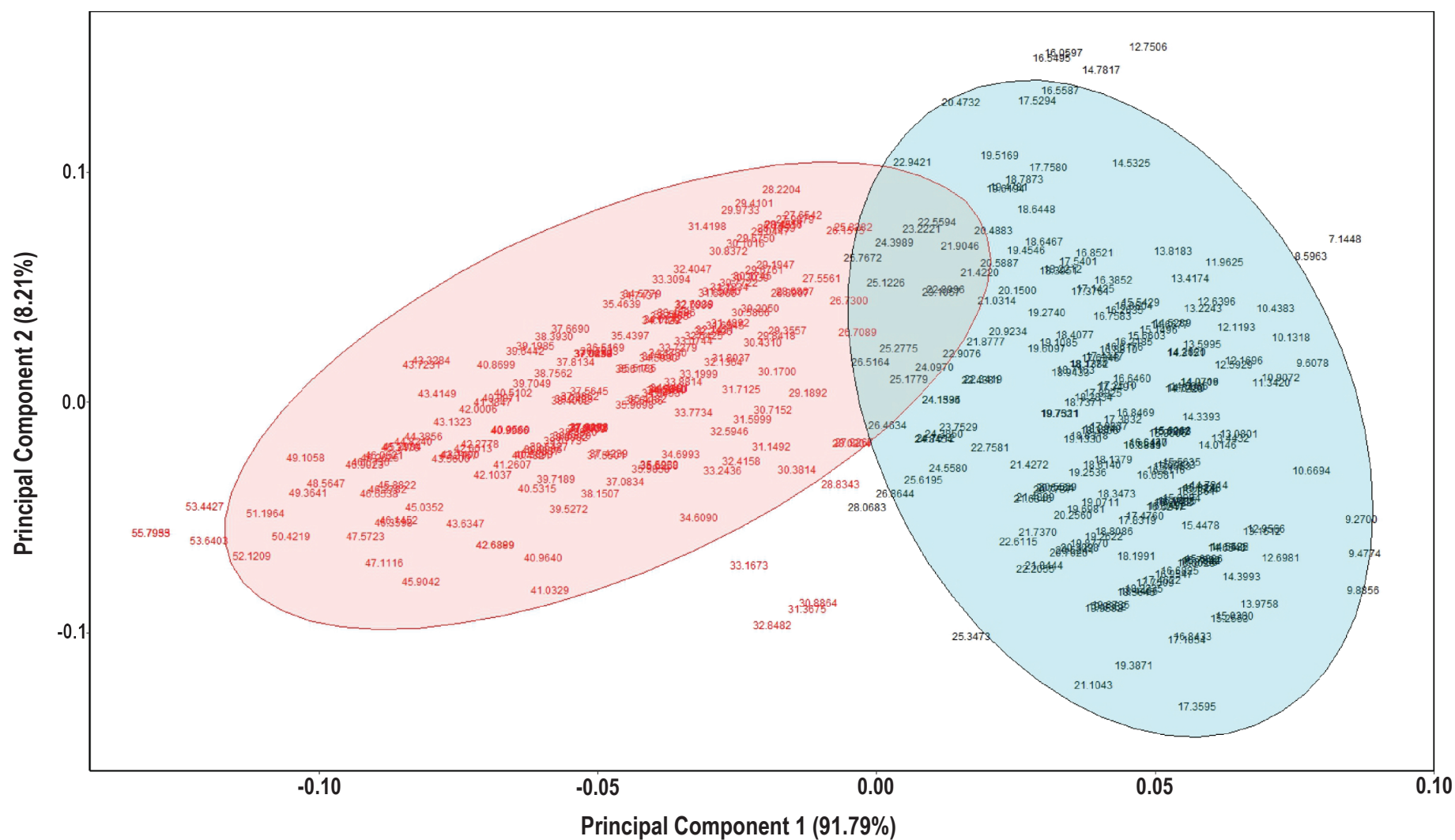

Supplement: S1 Fig — PCA analysis performed on areaP/diameterG and age of death (days) values of 387 wild-type animals from 8 separate experiments on OP50. Numerical values of datapoints represent the areaP/diameterG of each dead animal. Animals are separated into two clusters—probability ellipses shown in red (left side) and in blue (right side). The red cluster represents animals with swollen pharynges at death (P-deaths). The blue cluster represents animals with non-swollen pharynges at death (non-P deaths). (PDF) [file pgen.1012190.s001.pdf]

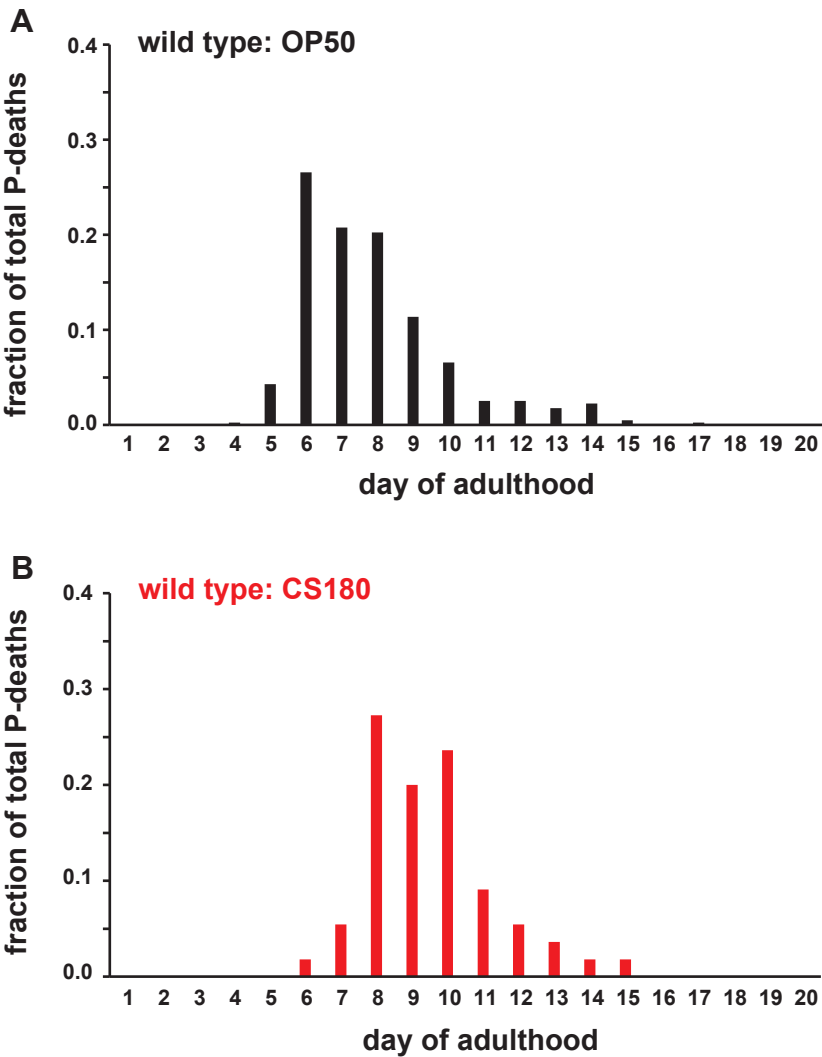

Supplement: S2 Fig — (A-B) P-deaths were no longer observed after day 15 of adulthood on OP50 (A) and on CS180 (B). The number of P deaths on OP50 was 395 out of 1070 deaths (number of trials, 10). The number of P deaths on CS180 was 55 out of 528 deaths (number of trials, 6). (PDF) [file pgen.1012190.s002.pdf]

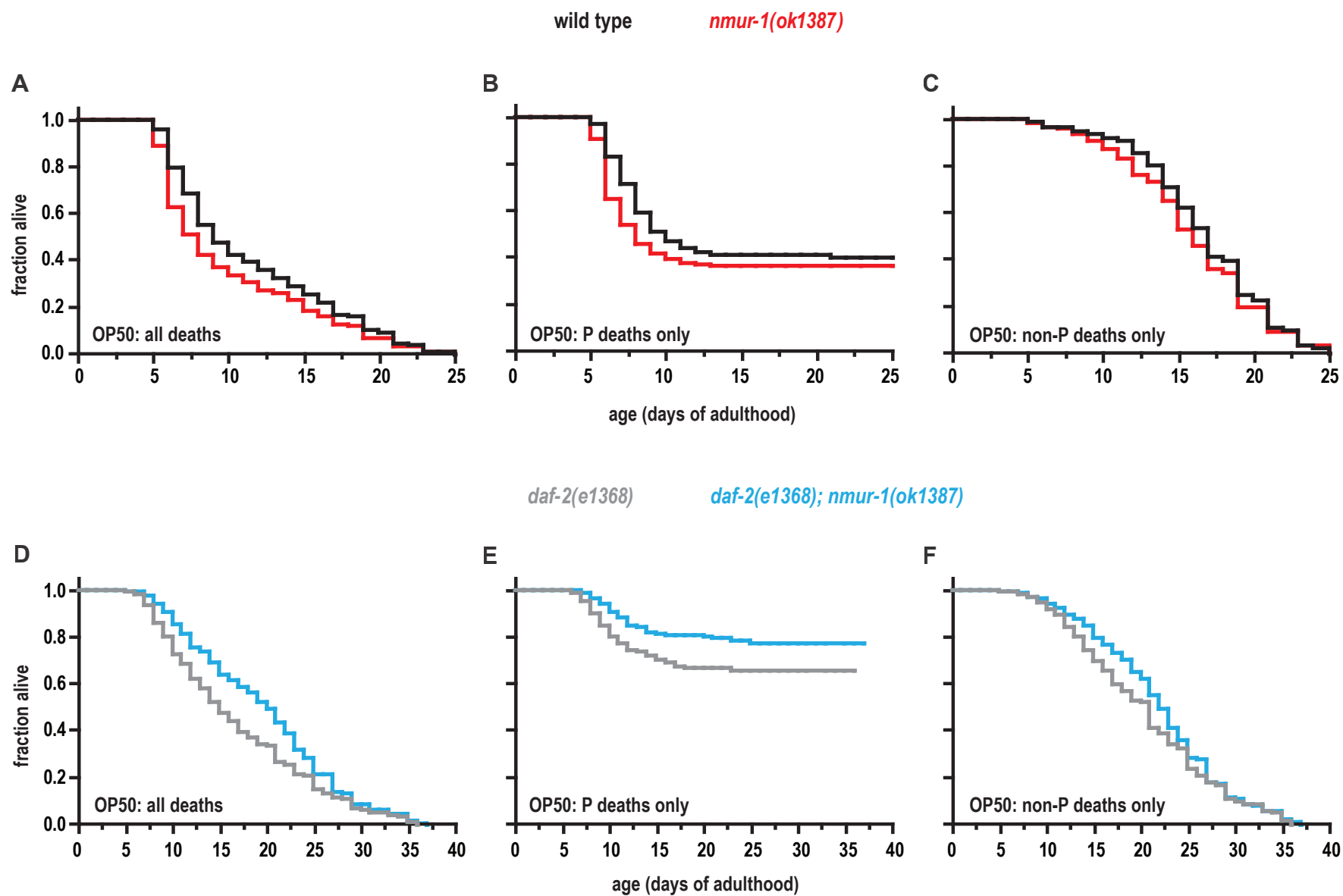

Supplement: S3 Fig — (A-C) The survival curves of wild type and nmur-1(ok1387) single mutants (cumulative of 7 independent trials from Figs 2, 3 and 7), when all types of deaths (A) or only P-deaths (B) are included or when P-deaths are excluded (C). (D-F) The survival curves of daf-2(e1368) single mutants and daf-2(e1368); nmur-1(ok1387) double mutants (cumulative of 6 independent trials from Figs 3 and 7), when all types of deaths (D) or only P-deaths (E) are included or when P-deaths are excluded (F). See S5 Table for the statistical analyses of these data. (PDF) [file pgen.1012190.s003.pdf]

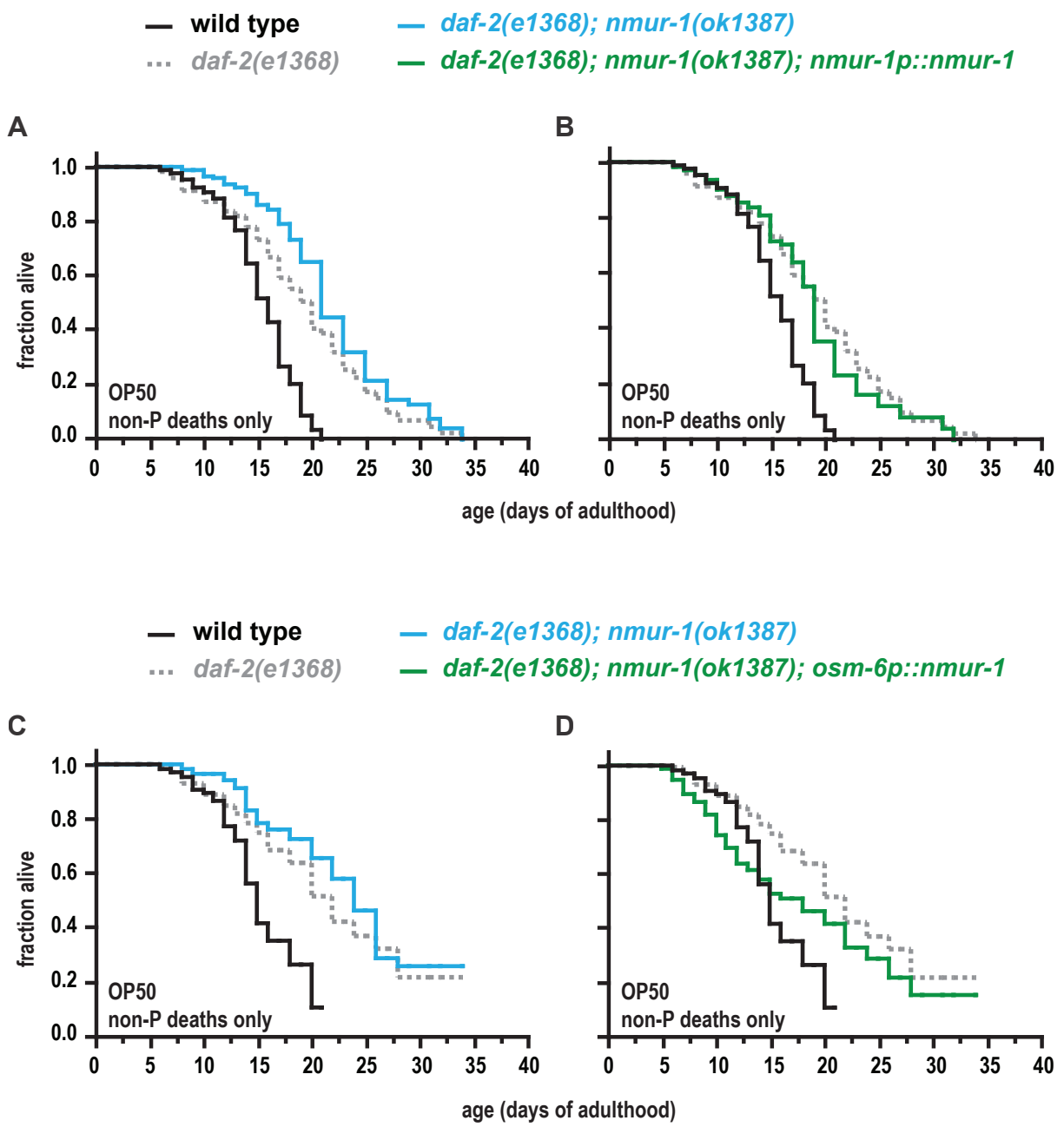

Supplement: S4 Fig — (A-D) The non-P death phenotypes of daf-2(e1368) single mutants versus daf-2(e1368); nmur-1(ok1387) double mutants (A, C), where nmur-1 was rescued in nmur-1-expressing cells (B) or in sensory neurons alone (D). See S5 Table for the statistical analyses of these data. (PDF) [file pgen.1012190.s004.pdf]
