# Supplementary material for: The neuropeptide neuromedin U receptor NMUR-1 buffers insulin receptor signaling in bacteria-dependent C. elegans survival
Source: PLoS Genet. 2026 Jun 11;22(6):e1012190. doi: 10.1371/journal.pgen.1012190 (PMC13289896; doi:10.1371/journal.pgen.1012190)
Supplement: S5 Table — Cumulative statistics of all types of deaths of the indicated C. elegans strains. P values that are significant (P ≤ 0.05) are italicized and in bold face. If the test population lived longer or had fewer P-deaths than the population to which it is compared, the P values are also underlined. The superscripts indicate the population to which the test population is compared. ok1387* indicates the genotype daf-2(e1368); nmur-1(ok1387). The symbol ** denotes that the pharyngeal sizes of late deaths were left unmeasured. (PDF) [file pgen.1012190.s009.pdf]

Table S5. *nmur-1*-dependent P-deaths versus non-P deaths on OP50

Strain	Mean Lifespan ± SEM (Days)	# Animals Observed/ Total Initial Animals (# Trials)	*P* vs specified group (Logrank)	*P* vs specified group (Wilcoxon)	Fig
		all deaths			
Wild type (WT)	11.3 ± 0.2	580/1052 (7)	-	-	S3A
nmur-1(ok1387)	10.1 ± 0.2	675/1052 (7)	***< 0.0001***^{WT}	***< 0.0001***^{WT}	S3A
daf-2(e1368)	16.8 ± 0.4	375/1112 (6)	-	-	S3D
daf-2(e1368); nmur-1(ok1387)	19.6 ± 0.4	350/892 (6)	***< 0.0001***$^{daf-2}$	***< 0.0001***$^{daf-2}$	S3D
		P deaths only			
Wild type (WT)		392/1052 (7)	-	-	S3B
nmur-1(ok1387)		481/1052 (7)	***< 0.0001***^{WT}	***< 0.0001***^{WT}	S3B
daf-2(e1368)		164/1112 (6)	-	-	S3E
daf-2(e1368); nmur-1(ok1387)		104/892 (6)	***< 0.0001***$^{daf-2}$	***< 0.0001***$^{daf-2}$	S3E
		non-P deaths only			
Wild type (WT)	16.7 ± 0.3	188/1052 (7)	-	-	S3C
nmur-1(ok1387)	15.9 ± 0.3	194/1052 (7)	0.08^{WT}	0.09^{WT}	S3C
daf-2(e1368)	20.4 ± 0.5	206/1112 (6)	-	-	S3F
daf-2(e1368); nmur-1(ok1387)	21.9 ± 0.4	244/892 (6)	***0.01***$^{daf-2}$	***0.004***$^{daf-2}$	S3F
		non-P deaths in the rescue experiments			
		Rescue of daf-2(e1368); nmur-1(ok1387) double mutants with nmur-1p::nmur-1			
Wild type (WT)	15.4 ± 0.3	90/416 (3)	-	-	S4A,B
daf-2(e1368)	19.3 ± 0.6	118/416 (3)	***< 0.0001***^{WT}	***0.05***^{WT}	S4A,B
daf-2(e1368); nmur-1(ok1387)	21.8 ± 0.6	92/496 (3)	***< 0.0001***^{WT}<br>***0.0004***$^{daf-2}$	***< 0.0001***^{WT}<br>***< 0.0001***$^{daf-2}$	S4A
daf-2(e1368); nmur-1(ok1387); *nmur-1p::nmur-1*	18.8 ± 0.5	112/496 (3)	***< 0.0001***^{WT}<br>0.6^{daf-2}<br>***< 0.0001***^{ok1387*}	***0.007***^{WT}<br>0.8^{daf-2}<br>***< 0.0001***^{ok1387*}	S4B
		Rescue of daf-2(e1368); nmur-1(ok1387) double mutants with osm-6p::nmur-1			
Wild type (WT)	15.3 ± 0.4**	66/336 (2)	-	-	S4C,D
daf-2(e1368)	20.7 ± 0.6**	71/336 (2)	***< 0.0001***^{WT}	***0.003***^{WT}	S4C,D
daf-2(e1368); nmur-1(ok1387)	22.3 ± 0.5**	73/336 (2)	***< 0.0001***^{WT}<br>***0.05***$^{daf-2}$	***< 0.0001***^{WT}<br>***0.006***$^{daf-2}$	S4C
daf-2(e1368); nmur-1(ok1387); *osm-6p::nmur-1*	17.8 ± 0.7**	88/336 (2)	0.5^{WT}<br>***0.002***$^{daf-2}$<br>***< 0.0001***^{ok1387*}	***0.04***^{WT}<br>***< 0.0001***$^{daf-2}$<br>***< 0.0001***^{ok1387*}	S4D
