# Supplementary material for: The neuropeptide neuromedin U receptor NMUR-1 buffers insulin receptor signaling in bacteria-dependent C. elegans survival
Source: PLoS Genet. 2026 Jun 11;22(6):e1012190. doi: 10.1371/journal.pgen.1012190 (PMC13289896; doi:10.1371/journal.pgen.1012190)
Supplement: Attachment — Submitted filename: _Sifoglu et al_Responses to Reviewers.pdf [file pgen.1012190.s018.pdf]

General comments:

We thank all the Reviewers for their constructive comments and have performed additional experiments and revised the manuscript according to their suggestions. We think that these revisions have greatly improved our manuscript. Our detailed responses to individual comments are indicated below in blue text.

Point-by-point Responses:

Comments to the Authors:

Please note here if the review is uploaded as an attachment.

Reviewer #1: This is a very nice paper by Deniz Sifoglu et al in Joy Alcedo's lab examining how the neuromedin U receptor regulates lifespan in the nematode C. elegans. They build on their previous work showing that nmur-1 regulates lifespan in a bacterial LPS-specific manner. Here they show that many of these effects are though modulation of one specific cause of death related to pharyngeal swelling. Interestingly, nmur-1 does not have a simple effect on lifespan, instead its effect on lifespan is dependent on the bacterial LPS. Even more interestingly, the type of influence of NMUR-1 on lifespan depends on the level of signaling by the insulin receptor DAF-2. At normal levels of DAF-2, NMUR-1 increases lifespan, but at low levels of DAF-2 (which increase lifespan), NMUR-1 shorten lifespan. Thus, as the authors conclude, it appears that NMUR-1 limits the dynamic range of the lifespan effects of DAF-2 by influencing both its lower and upper bounds. I think this is a conceptually important paper and , therefore, support its publication.

Response: We thank the Reviewer very much for these comments.

I have a series of minor suggestions that I think may make the paper easier to read to non-geneticists. The use of the word activity (eg "nmur-1 adjusts and buffers insulin receptor activity") can be quite un-intuitive to non-geneticists. I suppose by genetic activity they mean, how much a gene influences a phenotype (?), here lifespan and P death. But this influence on activity, confusingly, is not necessarily direct, it can be upstream or downstream of the gene function, and it is confusing if they refer about the activity of the DAF-2 protein or the daf-2 gene, or both. I understand that activity packs all these meanings in one word, but I suggest they unpack these terms because the readers may not know what they mean. Similarly, it would help if when they refer to the normal gene activity they wrote nmur-1(+) instead of nmur-1, to more explicitly differentiate it from the mutant allele.

Response: We thank the Reviewer for these suggestions. Because the *daf-2* mutations reduce DAF-2 protein activity (Gems et al, Genetics 1998, vol 150, pp 129-15; Patel et al, Genetics 2008, vol 178, pp 931-946), we clarified in the text that we are referring to protein activity. We also changed nmur-1 to nmur-1(+), when we are specifically referring to the wild-type allele in the main text.

The paper ends kind of with a teaser for future studies, showing that nmur-1 regulates the expression of daf-28/insulin. It would be nice if they also looked at whether the LPS-affecting E. coli mutants phenocopy those effects. But I don't think it is essential.

Response: We agree with the Reviewer that the regulation of the insulin-like peptide (ILP) *daf-28* by wild-type NMUR-1 provides a direction for future studies. In addition to our earlier findings that loss of *nmur-1* upregulates the expression of the ILP *daf-28* when DAF-2 has wild-type protein activity (now shown as Fig 7B), we tested the effect of *daf-28* on the number of P-deaths (Fig 7D). We find that loss of *daf-28* reduces P-deaths (Fig 7D). This is consistent with the model that wild-type NMUR-1 inhibits the expression of *daf-28*, a presumed DAF-2 agonist (Cornils et al, Development 2011, vol 138, pp 1183-1193; Fernandes de Abreu et al, PLoS Genet 2014, vol 10, e1004225), which in turn inhibits wild-type DAF-2 protein activity (Fig 8). Moreover, we show that loss of *nmur-1* has little or no effect on *daf-28* expression when DAF-2 has low protein activity due to a reduction-in-function point mutation (Fig 7C). This is also consistent with the model that wild-type NMUR-1 buffers the range of DAF-2 receptor activity by regulating the expression or activity of other ILPs (Fig 8).

In many figures the authors plot survival curves with "P-deaths only." I suppose that what they are doing is to censor the non-P deaths? In any case, they should explain in the methods what is it that they are doing to make those plots, and ideally in the main text.

Response: We have now written in the main text, in the methods and in the figure legends that we are censoring non-P deaths when we are assessing P death-dependent survival. We also stated in the manuscript that we are censoring P deaths, when we are assessing survival that only includes non-P deaths.

Reviewer #2: In this article by Sifoglu et al., the authors report that differences in C. elegans death on two different E. coli strains can be attributed to differences in bacterial LPS structures and nmur-1 activity. Mutants deficient in nmur-1 exhibit greater P-deaths on OP50 but not on CS180 bacteria. However, the authors make the surprising observation that nmur-1 and daf-2 double mutants are long-lived with fewer P-deaths than daf-2 single mutants. They determine that these phenotypes are dependent on daf-16, the downstream transcription factor of daf-2 signaling. This suggests that depending on the level of insulin signaling, nmur-1 can have opposing roles on P-deaths on OP50. Lastly, the authors demonstrate that rescuing nmur-1 activity in sensory neurons in nmnur-1 mutants rescues P-deaths on OP50. Overall, the findings of the paper and convincing, logical and rigorous and will appeal to a broad genetics audience. The data reveals a fascinating interaction between a host's genetic background, insulin signaling, and different bacterial food sources. Addressing the following concerns will strengthen these findings and the interpretation of the data:

Major concerns:
1) In figure 6A, the authors claim that nmur-1 mutants require daf-16 for P-body death. Both double mutants daf-16;nmur-1 and single mutants exhibit the same level of P-deaths, suggesting that daf-16 and nmur-1 are acting in the same pathway. However, this does not provide an order to claim that nmur-1 acts upstream of daf-16 as depicted in Figure 7.

Response: We thank the Reviewer for this comment. We addressed this by looking at the expression of the DAF-16 target gene sod-3 in wild type versus nmur-1 single mutant or daf-2 single mutant versus daf-2; nmur-1 double mutant. Accordingly, we found that expression of the sod-3p::GFP transcriptional reporter is significantly higher in daf-2; nmur-1 double mutants compared to daf-2 single mutants (Fig 7C; Table S12). This is consistent with our model that wild-type NMUR-1 upregulates the activity of a mutant DAF-2 receptor to inhibit DAF-16 activity (Fig 8, condition 2).

On the other hand, sod-3p::GFP levels are similar between wild type and nmur-1 single mutants, which would suggest that wild-type NMUR-1 restricts wild-type DAF-2 activity in parallel to DAF-16, which we have now also depicted in Fig 8 (condition 1). However, sod-3 is only activated by some isoforms of DAF-16 (Kwon et al, Nature 2010, vol 466, pp 498-502). Since the daf-16 null allele we used, mu86, deletes all isoforms (Kwon et al 2010), it remains a formal possibility that wild-type NMUR-1 also inhibits DAF-2 signaling through a specific isoform of DAF-16. Thus, we have placed a question mark on the dotted inhibitory arrows extending from wild-type DAF-2 in Fig 8 (condition 1).

2) Interestingly, reducing daf-2 (thereby activating daf-16) protects against death and nmur-1 loss-of-function mutants are further protected. This suggests that nmur-1 is acting in a parallel protective pathway with daf-16 in daf-2 mutants. In Figure 6B, the authors demonstrate that the triple mutant (daf-2, daf-16, and nmur-1) exhibit the same lifespan and P-deaths as single mutants. This suggests that daf-16 is the decisive node, because in daf-16 mutants, the level of daf-2 or nmur-1 does not change the outcome. Nmur-1 could be acting upstream of daf-16 or in parallel, but one cannot conclude which from the provided data. The authors should use a daf-16 reporter or a sod-3p::GFP reporter to determine if nmur-1 regulates daf-16 directly and if daf-16 activity changes in the daf-2;nmur-1 double mutant.

Response: Please see our response to point 1 above.

3) The authors show osm-6p::nmur-1; nmur-1 mutant animals have fewer P-body deaths. Is there increased daf-16 signaling in these animals?

Response: We thank the Reviewer for this interesting question, but we think it is beyond the scope of this manuscript. We think that these points will be best addressed by future experiments.

4) The authors show that daf-28 is increased in Fig 6D. However, there is no data demonstrating that daf-28 mutants suppress P-deaths induced by loss of nmur-1 on OP50 or that increasing daf-28 levels alone is sufficient to induce P body death on OP50. Elevated daf-28 could be correlation and the evidence does not exist to place it in the Figure 7 unless there is a ? mark after daf-28.

Response: We have now tested the effect of a daf-28 deletion mutant on P-deaths and found that there are fewer P deaths. Thus, this suggests that NMUR-1 inhibits daf-28 expression to limit wild-type DAF-2 receptor activity, and thus reduce P-deaths.

5) Figure 7 is very confusing. The use of "high activity" of daf-2 does not make sense to describe basal levels. To generate a high level of insulin/IGF signaling the authors should use gain of function mutants for pdk-1 or akt-1. I recommend the authors draw out the schematic for normal/wild-type conditions and the daf-2 mutant/reduced insulin signaling condition separately.

Response: We apologize for the confusion. We have now changed "high activity" to "wild-type function" and "low activity" to "reduction in function".

6) The authors claim that the data in Figure "suggests that wild-type nmur-1 in sensory neurons inhibits P-deaths when daf-2 activity is high but promotes P deaths under low daf-2 activity." However, the gray and green dotted lines indicating the daf-2 mutant and daf-2 mutant with wild-type nmur-1 in sensory neurons show similar levels of P-deaths (ie. not more p-deaths in green dotted line compared to gray dotted line). Also, stating "high" level of daf-2 suggests higher than normal/wild-type.

Response: We have clarified the role of wild-type NMUR-1 in sensory neurons. We now state the following:

"Expression of *nmur-1(+)* from the sensory neuron-specific promoter *osm-6p* [21] likewise rescued the *nmur-1* mutant survival phenotypes in wild-type and mutant *daf-2* backgrounds (Figs 4B, 4D, S4C and S4D; Tables 1, S5, S8 and S9). This result suggests that NMUR-1(+) in sensory neurons inhibits P-deaths when DAF-2 protein activity is wild type, although it has less of an effect on P-deaths when DAF-2 receptor activity is reduced (Fig 4B and 4D; Tables 1 and S9). Interestingly, NMUR-1(+) in sensory neurons rescued the non-P death phenotype of *daf-2(e1368); nmur-1(ok1387)* double mutants (Fig S4C and S4D; Tables S5 and S8) more robustly than the P-death phenotype of these animals (Fig 4D; Tables 1 and S9). Together these data suggest that wild-type NMUR-1 acts in sensory neurons to exert its multiple, context-dependent effects on *C. elegans* survival."

Minor concerns:
1. Some areas of the text have confusing wording. For example, "The same construct

rescued both the P-death and non-P death phenotypes…” makes it sound to the reader as though there are other phenotypes associated with P-death or non-P death that were assayed. I recommend, “The same construct rescued P-death and non-P death…”

Response: We apologize for the confusion. We have clarified some of the text that we think might cause some confusion. We also removed “both” from the sentence cited by the Reviewer.

2. The phrase “inhibits healthy pharynges” is nonsensical.

Response: We have changed this into “NMUR-1 decreases the number of healthy pharynges”.

3. It is not clear from the plot that 3F is the difference between the wild-type and daf-2 mutant background which could lead to misunderstanding if the legend is not read carefully. I suggest adding a title to the plot “Difference in Median (daf-2mutant-wild-type)” or something similar.

Response: We have added to the Y-axis in Fig 3F the description: “difference in median deaths of wild type versus *daf-2* mutants (days)”.

Reviewer #3: This manuscript provides strong genetic evidence for the function of nmur-1 in regulating P-death, both in the presence and absence of daf-2. Overall, the study is well written, and I have no major concerns regarding the experimental design or data interpretation.

Response: We thank the Reviewer very much for these comments.
