# Supplementary material for: The neuropeptide neuromedin U receptor NMUR-1 buffers insulin receptor signaling in bacteria-dependent C. elegans survival
Source: PLoS Genet. 2026 Jun 11;22(6):e1012190. doi: 10.1371/journal.pgen.1012190 (PMC13289896; doi:10.1371/journal.pgen.1012190)
Supplement: Peer review history

# Author comment: Author response to Decision Letter 0

**DOI:** 10.1371/journal.pgen.1012190.r001

**Transfer Alert**

This paper was transferred from another journal. As a result, its full editorial history (including decision letters, peer reviews and author responses) may not be present.

18 Aug 2025

# Peer review: Decision Letter 0

**DOI:** 10.1371/journal.pgen.1012190.r002

**Authors:** Fengwei Yu

16 Oct 2025

PGENETICS-D-25-00928

The neuropeptide neuromedin U receptor NMUR-1 buffers insulin receptor signaling in bacteria-dependent C. elegans survival

PLOS Genetics

Dear Dr. Alcedo,

Thank you for submitting your manuscript to PLOS Genetics. After careful consideration, we feel that it has merit but does not fully meet PLOS Genetics’s publication criteria as it currently stands. Therefore, we invite you to submit a revised version of the manuscript that addresses the points raised during the review process.

Please submit your revised manuscript within 60 days Dec 15 2025 11:59PM. If you will need more time than this to complete your revisions, please reply to this message or contact the journal office at plosgenetics@plos.org. When you’re ready to submit your revision, log on to https://www.editorialmanager.com/pgenetics/ and select the ‘Submissions Needing Revision’ folder to locate your manuscript file.

Please include the following items when submitting your revised manuscript:

* A rebuttal letter that responds to each point raised by the editor and reviewer(s). You should upload this letter as a separate file labeled ‘Response to Reviewers’. This file does not need to include responses to any formatting updates and technical items listed in the ‘Journal Requirements’ section below.

* A marked-up copy of your manuscript that highlights changes made to the original version. You should upload this as a separate file labeled ‘Revised Manuscript with Track Changes’.

* An unmarked version of your revised paper without tracked changes. You should upload this as a separate file labeled ‘Manuscript’.

If you would like to make changes to your financial disclosure, competing interests statement, or data availability statement, please make these updates within the submission form at the time of resubmission. Guidelines for resubmitting your figure files are available below the reviewer comments at the end of this letter.

We look forward to receiving your revised manuscript.

Kind regards,

Laura Bianchi

Academic Editor

PLOS Genetics

Fengwei Yu

Section Editor

PLOS Genetics

Aimée Dudley

Editor-in-Chief

PLOS Genetics

Anne Goriely

Editor-in-Chief

PLOS Genetics

**Journal Requirements:**

If the reviewer comments include a recommendation to cite specific previously published works, please review and evaluate these publications to determine whether they are relevant and should be cited. There is no requirement to cite these works unless the editor has indicated otherwise.

1) We ask that a manuscript source file is provided at Revision. Please upload your manuscript file as a .doc, .docx, .rtf or .tex. If you are providing a .tex file, please upload it under the item type u2018LaTeX Source Fileu2019 and leave your .pdf version as the item type u2018Manuscriptu2019.

2) Please provide an Author Summary. This should appear in your manuscript between the Abstract (if applicable) and the Introduction, and should be 150-200 words long. The aim should be to make your findings accessible to a wide audience that includes both scientists and non-scientists. Sample summaries can be found on our website under Submission Guidelines:

https://journals.plos.org/plosgenetics/s/submission-guidelines#loc-parts-of-a-submission

3) Please upload all main figures as separate Figure files in .tif or .eps format. For more information about how to convert and format your figure files please see our guidelines:

https://journals.plos.org/plosgenetics/s/figures

4) We notice that your supplementary Figures, and Tables are included in the manuscript file. Please remove them and upload them with the file type ‘Supporting Information’. Please ensure that each Supporting Information file has a legend listed in the manuscript after the references list.

5) We note that your Data Availability Statement is currently as follows: "All relevant data are within the manuscript and its Supporting Information files.". Please confirm at this time whether or not your submission contains all raw data required to replicate the results of your study. Authors must share the “minimal data set” for their submission. PLOS defines the minimal data set to consist of the data required to replicate all study findings reported in the article, as well as related metadata and methods (https://journals.plos.org/plosone/s/data-availability#loc-minimal-data-set-definition).

For example, authors should submit the following data:

1) The values behind the means, standard deviations and other measures reported;

2) The values used to build graphs;

3) The points extracted from images for analysis..

Authors do not need to submit their entire data set if only a portion of the data was used in the reported study.

If your submission does not contain these data, please either upload them as Supporting Information files or deposit them to a stable, public repository and provide us with the relevant URLs, DOIs, or accession numbers. For a list of recommended repositories, please see https://journals.plos.org/plosone/s/recommended-repositories.

If there are ethical or legal restrictions on sharing a de-identified data set, please explain them in detail (e.g., data contain potentially sensitive information, data are owned by a third-party organization, etc.) and who has imposed them (e.g., an ethics committee). Please also provide contact information for a data access committee, ethics committee, or other institutional body to which data requests may be sent. If data are owned by a third party, please indicate how others may request data access.

6)  Please ensure that the funders and grant numbers match between the Financial Disclosure field and the Funding Information tab in your submission form. Note that the funders must be provided in the same order in both places as well.

**Reviewers’ comments:**

Reviewer’s Responses to Questions

**Comments to the Authors:**

**Please note here if the review is uploaded as an attachment.**

Reviewer #1: This is a very nice paper by Deniz Sifoglu et al in Joy Alcedo’s lab examining how the neuromedin U receptor regulates lifespan in the nematode C. elegans. They build on their previous work showing that nmur-1 regulates lifespan in a bacterial LPS-specific manner. Here they show that many of these effects are though modulation of one specific cause of death related to pharyngeal swelling. Interestingly, nmur-1 does not have a simple effect on lifespan, instead its effect on lifespan is dependent on the bacterial LPS. Even more interestingly, the type of influence of NMUR-1 on lifespan depends on the level of signaling by the insulin receptor DAF-2. At normal levels of DAF-2, NMUR-1 increases lifespan, but at low levels of DAF-2 (which increase lifespan), NMUR-1 shorten lifespan. Thus, as the authors conclude, it appears that NMUR-1 limits the dynamic range of the lifespan effects of DAF-2 by influencing both its lower and upper bounds. I think this is a conceptually important paper and , therefore, support its publication.

I have a series of minor suggestions that I think may make the paper easier to read to non-geneticists. The use of the word activity (eg "nmur-1 adjusts and buffers insulin receptor activity") can be quite un-intuitive to non-geneticists. I suppose by genetic activity they mean, how much a gene influences a phenotype (?), here lifespan and P death. But this influence on activity, confusingly, is not necessarily direct, it can be upstream or downstream of the gene function, and it is confusing if they refer about the activity of the DAF-2 protein or the daf-2 gene, or both. I understand that activity packs all these meanings in one word, but I suggest they unpack these terms because the readers may not know what they mean. Similarly, it would help if when they refer to the normal gene activity they wrote nmur-1(+) instead of nmur-1, to more explicitly differentiate it from the mutant allele.

The paper ends kind of with a teaser for future studies, showing that nmur-1 regulates the expression of daf-28/insulin. It would be nice if they also looked at whether the LPS-affecting E. coli mutants phenocopy those effects. But I don’t think it is essential.

In many figures the authors plot survival curves with "P-deaths only." I suppose that what they are doing is to censor the non-P deaths? In any case, they should explain in the methods what is it that they are doing to make those plots, and ideally in the main text.

Reviewer #2: In this article by Sifoglu et al., the authors report that differences in C. elegans death on two different E. coli strains can be attributed to differences in bacterial LPS structures and nmur-1 activity. Mutants deficient in nmur-1 exhibit greater P-deaths on OP50 but not on CS180 bacteria. However, the authors make the surprising observation that nmur-1 and daf-2 double mutants are long-lived with fewer P-deaths than daf-2 single mutants. They determine that these phenotypes are dependent on daf-16, the downstream transcription factor of daf-2 signaling. This suggests that depending on the level of insulin signaling, nmur-1 can have opposing roles on P-deaths on OP50. Lastly, the authors demonstrate that rescuing nmur-1 activity in sensory neurons in nmnur-1 mutants rescues P-deaths on OP50. Overall, the findings of the paper and convincing, logical and rigorous and will appeal to a broad genetics audience. The data reveals a fascinating interaction between a host’s genetic background, insulin signaling, and different bacterial food sources. Addressing the following concerns will strengthen these findings and the interpretation of the data:

Major concerns:

1) In figure 6A, the authors claim that nmur-1 mutants require daf-16 for P-body death. Both double mutants daf-16;nmur-1 and single mutants exhibit the same level of P-deaths, suggesting that daf-16 and nmur-1 are acting in the same pathway. However, this does not provide an order to claim that nmur-1 acts upstream of daf-16 as depicted in Figure 7.

2) Interestingly, reducing daf-2 (thereby activating daf-16) protects against death and nmur-1 loss-of-function mutants are further protected. This suggests that nmur-1 is acting in a parallel protective pathway with daf-16 in daf-2 mutants. In Figure 6B, the authors demonstrate that the triple mutant (daf-2, daf-16, and nmur-1) exhibit the same lifespan and P-deaths as single mutants. This suggests that daf-16 is the decisive node, because in daf-16 mutants, the level of daf-2 or nmur-1 does not change the outcome. Nmur-1 could be acting upstream of daf-16 or in parallel, but one cannot conclude which from the provided data. The authors should use a daf-16 reporter or a sod-3p::GFP reporter to determine if nmur-1 regulates daf-16 directly and if daf-16 activity changes in the daf-2;nmur-1 double mutant.

3) The authors show osm-6p::nmur-1; nmur-1 mutant animals have fewer P-body deaths. Is there increased daf-16 signaling in these animals?

4) The authors show that daf-28 is increased in Fig 6D. However, there is no data demonstrating that daf-28 mutants suppress P-deaths induced by loss of nmur-1 on OP50 or that increasing daf-28 levels alone is sufficient to induce P body death on OP50. Elevated daf-28 could be correlation and the evidence does not exist to place it in the Figure 7 unless there is a ? mark after daf-28.

5) Figure 7 is very confusing. The use of “high activity” of daf-2 does not make sense to describe basal levels. To generate a high level of insulin/IGF signaling the authors should use gain of function mutants for pdk-1 or akt-1. I recommend the authors draw out the schematic for normal/wild-type conditions and the daf-2 mutant/reduced insulin signaling condition separately.

6) The authors claim that the data in Figure “suggests that wild-type nmur-1 in sensory neurons inhibits P-deaths when daf-2 activity is high but promotes P deaths under low daf-2 activity.” However, the gray and green dotted lines indicating the daf-2 mutant and daf-2 mutant with wild-type nmur-1 in sensory neurons show similar levels of P-deaths (ie. not more p-deaths in green dotted line compared to gray dotted line). Also, stating “high” level of daf-2 suggests higher than normal/wild-type.

Minor concerns:

1. Some areas of the text have confusing wording. For example, “The same construct rescued both the P-death and non-P death phenotypes…” makes it sound to the reader as though there are other phenotypes associated with P-death or non-P death that were assayed. I recommend, “The same construct rescued P-death and non-P death...”

2. The phrase “inhibits healthy pharynges” is nonsensical.

3. It is not clear from the plot that 3F is the difference between the wild-type and daf-2 mutant background which could lead to misunderstanding if the legend is not read carefully. I suggest adding a title to the plot “Difference in Median (daf-2mutant-wild-type)” or something similar.

Reviewer #3: This manuscript provides strong genetic evidence for the function of nmur-1 in regulating P-death, both in the presence and absence of daf-2. Overall, the study is well written, and I have no major concerns regarding the experimental design or data interpretation.

**********

**Have all data underlying the figures and results presented in the manuscript been provided?**

Large-scale datasets should be made available via a public repository as described in the *PLOS Genetics*  data availability policy, and numerical data that underlies graphs or summary statistics should be provided in spreadsheet form as supporting information.

Reviewer #1: Yes

Reviewer #2: Yes

Reviewer #3: Yes

**********

PLOS authors have the option to publish the peer review history of their article (what does this mean?). If published, this will include your full peer review and any attached files.

If you choose “no”, your identity will remain anonymous but your review may still be made public.

**Do you want your identity to be public for this peer review?**  For information about this choice, including consent withdrawal, please see our Privacy Policy.

Reviewer #1: No

Reviewer #2: No

Reviewer #3: No

**Figure resubmission:**

While revising your submission, we strongly recommend that you use PLOS’s NAAS tool (https://ngplosjournals.pagemajik.ai/artanalysis) to test your figure files. NAAS can convert your figure files to the TIFF file type and meet basic requirements (such as print size, resolution), or provide you with a report on issues that do not meet our requirements and that NAAS cannot fix.

After uploading your figures to PLOS’s NAAS tool - https://ngplosjournals.pagemajik.ai/artanalysis, NAAS will process the files provided and display the results in the "Uploaded Files" section of the page as the processing is complete. If the uploaded figures meet our requirements (or NAAS is able to fix the files to meet our requirements), the figure will be marked as "fixed" above. If NAAS is unable to fix the files, a red "failed" label will appear above. When NAAS has confirmed that the figure files meet our requirements, please download the file via the download option, and include these NAAS processed figure files when submitting your revised manuscript.

**Reproducibility:**

To enhance the reproducibility of your results, we recommend that authors of applicable studies deposit laboratory protocols in protocols.io, where a protocol can be assigned its own identifier (DOI) such that it can be cited independently in the future. Additionally, PLOS ONE offers an option to publish peer-reviewed clinical study protocols. Read more information on sharing protocols at https://plos.org/protocols?utm_medium=editorial-email&utm_source=authorletters&utm_campaign=protocols

# Author comment: Author response to Decision Letter 1

**DOI:** 10.1371/journal.pgen.1012190.r003

16 Apr 2026

Attachment Submitted filename: _Sifoglu et al_Responses to Reviewers.pdf

# Peer review: Decision Letter 1

**DOI:** 10.1371/journal.pgen.1012190.r004

**Authors:** Fengwei Yu

28 May 2026

Dear Dr Alcedo,

We are pleased to inform you that your manuscript entitled "The neuropeptide neuromedin U receptor NMUR-1 buffers insulin receptor signaling in bacteria-dependent C. elegans survival" has been editorially accepted for publication in PLOS Genetics. Congratulations!

Before your submission can be formally accepted and sent to production you will need to complete our formatting changes, which you will receive in a follow up email. Please be aware that it may take several days for you to receive this email; during this time no action is required by you. Please note: the accept date on your published article will reflect the date of this provisional acceptance, but your manuscript will not be scheduled for publication until the required changes have been made.

Once your paper is formally accepted, an uncorrected proof of your manuscript will be published online ahead of the final version, unless you’ve already opted out via the online submission form. If, for any reason, you do not want an earlier version of your manuscript published online or are unsure if you have already indicated as such, please let the journal staff know immediately at plosgenetics@plos.org.

In the meantime, please log into Editorial Manager at https://www.editorialmanager.com/pgenetics/, click the "Update My Information" link at the top of the page, and update your user information to ensure an efficient production and billing process. Note that PLOS requires an ORCID iD for all corresponding authors. Therefore, please ensure that you have an ORCID iD and that it is validated in Editorial Manager. To do this, go to ‘Update my Information’ (in the upper left-hand corner of the main menu), and click on the Fetch/Validate link next to the ORCID field.  This will take you to the ORCID site and allow you to create a new iD or authenticate a pre-existing iD in Editorial Manager.

If you have a press-related query, or would like to know about making your underlying data available (as you will be aware, this is required for publication), please see the end of this email. If your institution or institutions have a press office, please notify them about your upcoming article at this point, to enable them to help maximise its impact. Inform journal staff as soon as possible if you are preparing a press release for your article and need a publication date.

Thank you again for supporting open-access publishing; we are looking forward to publishing your work in PLOS Genetics!

Yours sincerely,

Laura Bianchi

Academic Editor

PLOS Genetics

Fengwei Yu

Section Editor

PLOS Genetics

Aimée Dudley

Editor-in-Chief

PLOS Genetics

Anne Goriely

Editor-in-Chief

PLOS Genetics

www.plosgenetics.org

BlueSky: @plos.bsky.social

----------------------------------------------------

Comments from the reviewers (if applicable):

Reviewer’s Responses to Questions

**Comments to the Authors:**

**Please note here if the review is uploaded as an attachment.**

Reviewer #1: I want to thank the authors for addressing the issues I raised (as well as those by the other reviewers). As with my previous recommendation, I think the paper should be accepted.

I have to say that I still find a lot of jargon in the paper very confusing. For example "wild type" is used as a genotype [eg daf-2(+)], that much is clear and is a very standard use. But in a lot of contexts it is also used to imply how the level of expression of a protein (or gene) in wild type animals under specific experimental conditions affects a phenotype [eg "Because wild-type DAF-28 increases P-deaths"]. This is confusing for several reasons, but especially because if the level of protein changes as part of the life of a wild type animal due to some sort of developmental or environmental or tissue-specific input, all of those levels are by definition "wild type". So many wild type levels are possible and normal. I would suggest that rather than trying to define what the right wild type level is, that the authors just refer to the level of protein or gene function that is occurring in the context they describe, relative to a context they define as a reference: for example none, low, medium/normal, high, very high.

Reviewer #2: The authors have addressed all substantive concerns in a thorough manner. The revisions have improved the clarity, rigor, and overall quality of the work, and I support its publication in PLOS Genetics.

Reviewer #3: Congratulations on the beautifully work! All concerns addressed!

**********

**Have all data underlying the figures and results presented in the manuscript been provided?**

Large-scale datasets should be made available via a public repository as described in the *PLOS Genetics*  data availability policy, and numerical data that underlies graphs or summary statistics should be provided in spreadsheet form as supporting information.

Reviewer #1: Yes

Reviewer #2: Yes

Reviewer #3: Yes

**********

PLOS authors have the option to publish the peer review history of their article (what does this mean?). If published, this will include your full peer review and any attached files.

If you choose “no”, your identity will remain anonymous but your review may still be made public.

**Do you want your identity to be public for this peer review?**  For information about this choice, including consent withdrawal, please see our Privacy Policy.

Reviewer #1: No

Reviewer #2: No

Reviewer #3: No

----------------------------------------------------

**Data Deposition**

If you have submitted a Research Article or Front Matter that has associated data that are not suitable for deposition in a subject-specific public repository (such as GenBank or ArrayExpress), one way to make that data available is to deposit it in the Dryad Digital Repository. As you may recall, we ask all authors to agree to make data available; this is one way to achieve that. A full list of recommended repositories can be found on our website.

The following link will take you to the Dryad record for your article, so you won’t have to re‐enter its bibliographic information, and can upload your files directly:

http://datadryad.org/submit?journalID=pgenetics&manu=PGENETICS-D-25-00928R1

More information about depositing data in Dryad is available at http://www.datadryad.org/depositing. If you experience any difficulties in submitting your data, please contact help@datadryad.org for support.

Additionally, please be aware that our data availability policy requires that all numerical data underlying display items are included with the submission, and you will need to provide this before we can formally accept your manuscript, if not already present.

----------------------------------------------------

**Press Queries**

If you or your institution will be preparing press materials for this manuscript, or if you need to know your paper’s publication date for media purposes, please inform the journal staff as soon as possible so that your submission can be scheduled accordingly. Your manuscript will remain under a strict press embargo until the publication date and time. This means an early version of your manuscript will not be published ahead of your final version. PLOS Genetics may also choose to issue a press release for your article. If there’s anything the journal should know or you’d like more information, please get in touch via plosgenetics@plos.org.

# Editor report: Acceptance letter

**DOI:** 10.1371/journal.pgen.1012190.r005

**Authors:** Fengwei Yu

PGENETICS-D-25-00928R1

The neuropeptide neuromedin U receptor NMUR-1 buffers insulin receptor signaling in bacteria-dependent C. elegans survival

Dear Dr Alcedo,

We are pleased to inform you that your manuscript entitled "The neuropeptide neuromedin U receptor NMUR-1 buffers insulin receptor signaling in bacteria-dependent C. elegans survival" has been formally accepted for publication in PLOS Genetics! Your manuscript is now with our production department and you will be notified of the publication date in due course.

The corresponding author will soon be receiving a typeset proof for review, to ensure errors have not been introduced during production. Please review the PDF proof of your manuscript carefully, as this is the last chance to correct any errors. Please note that major changes, or those which affect the scientific understanding of the work, will likely cause delays to the publication date of your manuscript.

Soon after your final files are uploaded, unless you have opted out or your manuscript is a front-matter piece, the early version of your manuscript will be published online. The date of the early version will be your article’s publication date. The final article will be published to the same URL, and all versions of the paper will be accessible to readers.

For Research Articles, you will receive an invoice from PLOS for your publication fee after your manuscript has reached the completed accept phase. If you receive an email requesting payment before acceptance or for any other service, this may be a phishing scheme. Learn how to identify phishing emails and protect your accounts at https://explore.plos.org/phishing.

Thank you again for supporting PLOS Genetics and open-access publishing. We are looking forward to publishing your work!

With kind regards,

Livia Horvath

PLOS Genetics

On behalf of:

The PLOS Genetics Team

Carlyle House, Carlyle Road, Cambridge CB4 3DN | United Kingdom

plosgenetics@plos.org | +44 (0) 1223-442823

plosgenetics.org | Twitter: @PLOSGenetics
